# Traumatic Cochlear Implant Electrode Extrusion: Considerations, Management, and Outcome

**DOI:** 10.1155/2021/2918859

**Published:** 2021-07-30

**Authors:** Ching Yee Chan, Feifan Wang, Haryani Omar, Henry Kun Kiaang Tan

**Affiliations:** ^1^Department of Otolaryngology, KK Women's and Children's Hospital, Singapore 229899; ^2^Audiology Service, KK Women's and Children's Hospital, Singapore 229899

## Abstract

Cochlear implantation is the mainstay for patients with severe to profound hearing loss that do not benefit from hearing aids. Falls and head trauma can cause direct damage to the implant, of which hard failure is the most common complication. Traumatic electrode migration is an uncommon occurrence. Our patient underwent successful electrode advancement of a partially migrated, normal functioning electrode two months after head injury. We discuss the factors influencing the decision-making process, progress, and outcomes.

## 1. Introduction

Childhood activities, changing levels of maturity, and motor skill development predispose children to head trauma. Falls, in particular, make up almost half of all head injuries in children [1]. Pediatric cochlear implant (CI) recipients are no exception. In fact, they may be at higher fall risk and subsequent head trauma from congenital hearing loss-associated vestibular dysfunction [2] or acquired vestibular dysfunction after the CI [3]. While the literature is present on hard failure after head trauma [4], detailed management plans are lacking. We share our experience with a case, the management, and eventual outcome.

## 2. Case Presentation

This is a brief report of one of our patients who received bilateral CI two years ago. Parental consent has been obtained. Case reports are exempt from Institutional Board Review in our institution. Our patient is a developmentally normal, four-year-old boy with bilateral profound congenital hearing loss who received bilateral CI (CI522, Cochlear Limited) via the round window technique at the age of 21 months. He presented a day after suffering a fall where he hit the left temporal parietal region over furniture. There was no open wound or bleeding, but patient was seen to have some bruising over the scalp at the area of injury. Parents sought medical attention when they noticed the patient removed the external processor each time the implant was switched on. He was able to communicate and hear because the right implant was unaffected. There were no other external injuries, and his conscious level was normal. The neurological and cranial nerve examination was intact. Both tympanic membranes were intact with air-filled middle ears. There was no rhinorrhoea, nystagmus, past pointing, or other signs of vestibular insufficiency. The gait was normal, and he was able to stand on one foot for five seconds.

**Figure 1:**
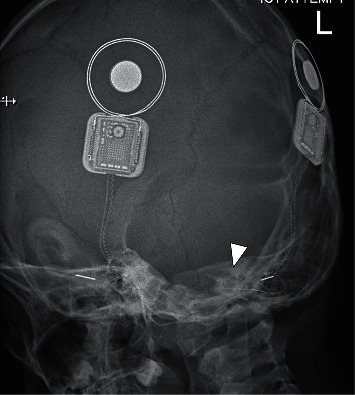
Lateral skull X-ray after trauma. The arrowhead points to the electrode which is curled in a 200-degree position. Comparing this with Figure 2 shows obvious migration of the electrode.

**Figure 2:**
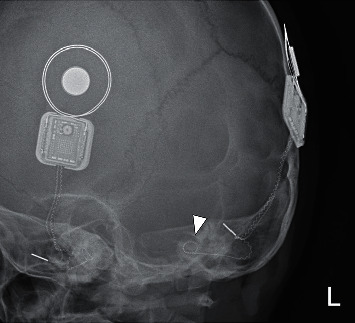
Lateral skull X-ray after initial cochlear implantation. The arrowhead points to the electrode which is curled in a 360-degree position, indicating its position in the basal and middle turn.

**Figure 3:**
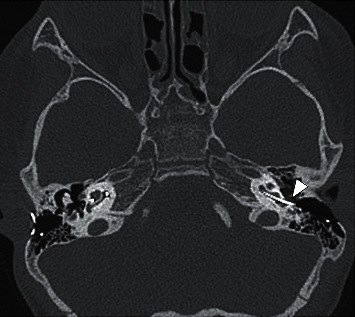
Computed tomography scan of the temporal bone with the arrowhead pointing to the electrodes outside the cochlear.

The patient was admitted for further evaluation. Neural response telemetry (NRT) performed showed 2 functioning midsection electrodes and normal impedance. Skull X-rays (Figure 1) showed displacement of the left implant electrode when compared to the post-CI X-ray (Figure 2). Computed tomography (CT) of the temporal bone showed ten of 22 electrodes out of the cochlear (Figure 3). The rest of the electrodes were in the basal turn with a few in the middle turn. Integrity testing of the implant was performed and indicated a normal functioning implant. Mapping was readjusted, and eight electrodes were switched off. The patient tolerated wearing the implant again. While the remap achieved hearing within the speech range, it was observed that his hearing performance deteriorated if the CI was worn on the injured side alone.

**Figure 4:**
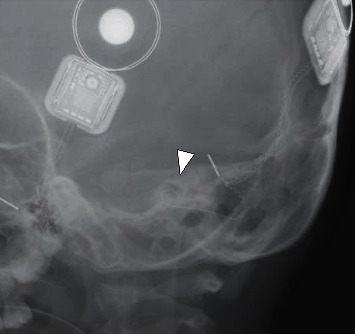
This is an intraoperative lateral skull X-ray taken after the electrode was advanced. The electrode resumed a similar position to Figure 2.

The patient was given the option of keeping the implant in the new position or undergoing surgical exploration and possible reinsertion of the implant. They decided on surgery, and this was performed 54 days after the date of injury. The patient underwent left revision mastoidectomy, exploratory tympanotomy, and reinsertion of CI under general anaesthesia. Intraoperatively, we found adhesions in the tympanic cavity around the electrode, which was partially extruded with both white markings in the tympanic cavity. The posterior tympanotomy appeared widened, suggesting shearing forces in this region leading to partial dislodgement of the electrode. We first performed adhesiolysis of the middle ear and then advanced the electrode smoothly back through the round window with jeweller's forceps and intraoperative X-rays and NRT yielded a good result (Figure 4). He was discharged after one day's observation, and switch on was performed three weeks later. All electrodes were working, and the aided thresholds were within speech range. A new map was created, and parents reported that the left hearing performance was back to baseline if only the left implant was switched on.

## 3. Discussion

Known CI complications include infection, device failure, tympanic membrane, and middle ear pathology such as tympanic membrane perforation [5]. Electrode migration is rare (0.7%), and these patients often present with gradual poor performance [4].

Head trauma is more often associated with device failure needing explantation than partial implant extrusion. The management of traumatic partial CI electrode extrusion has not been reported. Having performed close to 200 CI in the past decade, this is the first case we have encountered and we share our considerations.

1. Endolymph leak from electrode movement: we pack the round window edge around the electrode shaft with muscle and fascia during the CI. Scarring creates a natural plug which was dislodged when the electrode migrated outwards. Cerebrospinal fluid rhinorrhoea and middle ear effusion should be searched for. It is prudent to give antibiotics empirically to prevent meningitis, especially if the skin is breached, as this gives infection direct entry to the inner ear.
2. Endolymph disturbance can cause temporary vestibular disturbances, which should resolve with time. Meanwhile, the patient should be examined for imbalance and may need fall precautions.
3. The integrity and position of the electrode and patient hearing status: device failure necessitates explantation and replacement. A functioning, completely extruded device requires reimplantation. Depending on the presence of infection in the middle ear, inserting a new electrode may be preferred as there can be colonization of bacteria around the old electrode [6].
4. A functioning, incompletely extruded device requires advancement, which was performed in this scenario. The dilemma comes if the device is functioning but cannot be advanced. Should it be left in place, or removed, and reinserted, risking insertion failure? The decision to leave it or remove it and attempt reinsertion depends on the number of remnant electrodes in the cochlear—whether the new aided thresholds are satisfactory and the degree of residual hearing. As most patients have more severe high frequency than low frequency loss, electrodes in the basal turn can be adequate for amplification, and it may not be worthwhile risking losing the implant altogether. This brings us to the next point.
5. Status of the scala tympani: scala fibrosis can occur after CI [7] and should be monitored before surgery. CT scan gives a clue but has limited sensitivity and specificity [8]. MRI is less useful because the magnet results in a large imaging defect in the area of interest. Removing the magnet will require a separate surgical procedure. This reduces the size of the artefact but potentially still affects the area of interest [9]. If part of the implant is in situ, measuring impedance over time may be a useful tool as increasing impedance suggests cochlear obliteration [10]. While increasing impedance should prompt earlier surgery, stable impedance does not mean the converse because impedance can only measure the portion of the cochlear where electrodes are in contact with. It does not assess the middle turn cochlear where the reinsertion will take place.
6. In total extrusion, attempts to overcome scala fibrosis can be made using techniques described to overcome *Labyrinthine ossificans*, such as using a CI with a stylet, basal turn drill out, and scala vestibuli insertion. In incomplete extrusion, these approaches may be less favourable than leaving the existing CI in place, and a decision can be made based on the discussion in point 3. The possibility of complete loss of hearing (if the implant is removed and cannot be reinserted) and extra cost with a new implant should be emphasised and an informed choice is made.
7. Timing of surgery: the NRT performed the day after injury showed two functioning electrodes. Two days later, the NRT showed all electrodes were functioning. The integrity test two weeks later was also normal. This case shows there can be transient disruption to electrode function with spontaneous recovery. Because of this, we recommend serial testing after traumatic extrusion until normal function is achieved or until function stabilises. In a patient with bilateral hearing loss and a single sided implant, timing is balanced with the severity of suboptimal hearing.
8. Theoretically, electrode avulsion is a traumatic event and may induce inflammation and fibrosis. In this context, early intervention is necessary to prevent failure of reinsertion. While we are unable to recommend an exact time frame, we can extrapolate data from postmeningitis labyrinthine fibrosis, which occurs as early as four weeks after the infection [8]. Our patient underwent surgery two months from the accident, and the reinsertion was smooth. We thus recommend two months as a favourable timeframe, until further reports yield more results.
9. Follow-up: device failure can occur following head trauma and may not be immediately apparent [4]. The patient should be observed for poor CI performance and repeat integrity testing performed if necessary.

### 3.1. Future Directions

This is the only case of traumatic CI extrusion we have encountered. Reported cases of spontaneous electrode migration are with straight implants [6]. Shifting preferences towards perimodiolar implants may further reduce this occurrence in future. Several ways to secure the implant have been described [11, 12], though depending on the force of the trauma, the tie may not be able to hold the electrode in place.

Our centre has moved towards bilateral simultaneous CI since 2019. This allows the patient to hear and communicate even if one implant malfunctions. Where funding permits, patients with bilateral severe hearing loss should have bilateral CI.

## Data Availability

As this is a case report on a patient's clinical progress, data collection was unnecessary. Further imaging details are available from the corresponding author upon request.
